# Development of Novel Immunotherapies for Multiple Myeloma

**DOI:** 10.3390/ijms17091506

**Published:** 2016-09-08

**Authors:** Ensaf M. Al-Hujaily, Robyn A. A. Oldham, Parameswaran Hari, Jeffrey A. Medin

**Affiliations:** 1 Department of Pediatrics, Medical College of Wisconsin, Milwaukee, WI 53226, USA; ealhejaily@gmail.com (E.M.A.-H.); robyn.oldham@mail.utoronto.ca (R.A.A.O.); 2 Department of Medical Biophysics, University of Toronto, Toronto, ON M5G 1L7, Canada; 3 Department of Medicine, Division of Hematology/Oncology, Medical College of Wisconsin, Milwaukee, WI 53226, USA; phari@mcw.edu; 4 The Institute of Medical Sciences, University of Toronto, Toronto, ON M5S 1A8, Canada; 5 Department of Biochemistry, Medical College of Wisconsin, Milwaukee, WI 53226, USA; 6 Cancer Center, Medical College of Wisconsin, Milwaukee, WI 53226, USA

**Keywords:** multiple myeloma, monoclonal antibodies, stem cell transplantation, CAR-T cell therapy

## Abstract

Multiple myeloma (MM) is a disorder of terminally differentiated plasma cells characterized by clonal expansion in the bone marrow (BM). It is the second-most common hematologic malignancy. Despite significant advances in therapeutic strategies, MM remains a predominantly incurable disease emphasizing the need for the development of new treatment regimens. Immunotherapy is a promising treatment modality to circumvent challenges in the management of MM. Many novel immunotherapy strategies, such as adoptive cell therapy and monoclonal antibodies, are currently under investigation in clinical trials, with some already demonstrating a positive impact on patient survival. In this review, we will summarize the current standards of care and discuss major new approaches in immunotherapy for MM.

## 1. Introduction

Multiple myeloma (MM) accounts for 13% of all hematological malignancies, with an annual incidence of 6.5 per 100,000 people in the Western world [1,2]. MM is characterized by clonal expansion of malignant plasma cells in bone marrow (BM) resulting in excessive production of monoclonal immunoglobulins in the serum and/or the urine [1,3]. MM is a multistep progressive disorder that arises from the pre-malignant proliferation of plasma cells. This initial benign condition is referred to as monoclonal gammopathy of undetermined significance (MGUS). MGUS may progress to an asymptomatic (smoldering) myeloma and eventually to symptomatic MM. Both smoldering myeloma and MM are characterized by clonal expansion of malignant plasma cells in the BM and production of paraproteins (monoclonal immunoglobulin). However, MM is differentiated from smoldering myeloma by the development of clinical symptoms, such as hypercalcemia, renal insufficiency, anemia, and bone disease (often described by the acronym CRAB) or biomarkers predicting imminent development of CRAB [3,4].

Progression of MM involves several genetic and epigenetic abnormalities of the plasma cells, accompanied by changes in the BM microenvironment [4,5,6]. Genetic abnormalities result from a combination of gains and losses of chromosomal regions by non-random chromosomal translocations and by point mutations [7,8,9]. These include activation of oncogenes, such as *MYC* [10], *NRAS*, *KRAS* [11,12], and fibroblast growth factor receptor-3 (FGFR-3) [9]. Mutations also cause loss of the tumor suppressor protein *TP53* [13] and inactivation of cyclin-dependent kinase inhibitors, *CDKN2A* and *CDKN2C* [14]. Other abnormalities involve epigenetic dysregulation, such as modifications in gene methylation [15] and alterations in microRNA expression [16]. These abnormalities play a key role in determining tumor progression and drug resistance as they alter responses to growth stimuli in the microenvironment, as well as the expression of adhesion molecules on myeloma cells [1,4,17].

**Figure 1:**
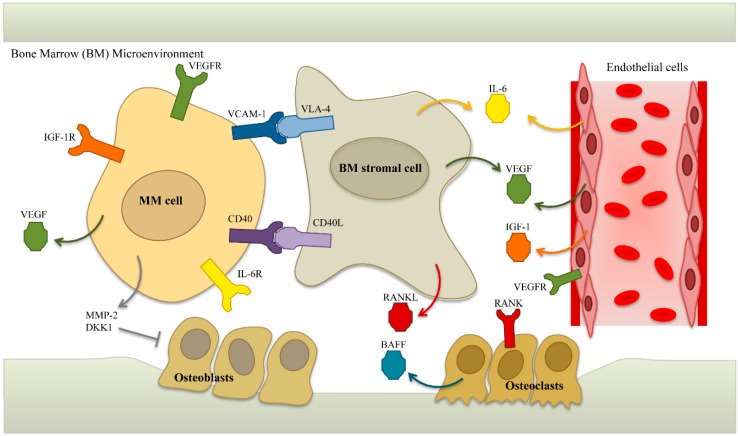
Interactions between multiple myeloma (MM) cells and the bone marrow (BM) niche. Adhesion of MM cells to BM stromal cells is mediated by cell-adhesion molecules including vascular cell adhesion molecule-1 (VCAM-1) and integrin α-4 (VLA-4). This adhesion triggers secretion of cytokines, such as VEGF and IL-6, from both MM cells and BM stromal cells. Both of these cytokines stimulate the growth of MM cells and development of the neo-vasculature. Endothelial cells, in turn, secrete more VEGF, IL-6, and IGF-1, further enhancing growth and survival of MM cells. Furthermore, receptor activator of NFκB ligand (RANKL) is produced by BM stromal cells and stimulates osteoclastogenesis. In contrast, osteoblast differentiation is inhibited by Dickkopf-1 (DKK-1), which is produced by MM cells. MM cells also secrete metalloproteases, such as MMP-2, resulting in degradation of the BM niche. While inhibition of osteoblastogenesis promotes osteolysis, degradation of the BM environment further enhances homing of the MM cells.

Adhesion of MM cells to BM stromal cells stimulates tumor cell proliferation and anti-apoptotic pathways [1,17,18]. As seen in Figure 1, MM cells may also produce growth factors such as vascular endothelial growth factor (VEGF), basic fibroblast growth factor (bFGF), and hepatocyte growth factor (HGF), which stimulate angiogenesis [19,20]. Angiogenesis promotes MM growth in the BM by increasing the delivery of oxygen and nutrients, and through the associated secretion of growth factors such as interleukin (IL)-6 and insulin-like growth factor-1 (IGF-1), by endothelial cells, both of which are potent growth factors for MM cells [21,22,23]. Furthermore, BM stromal cells secrete IL-8, which allows MM cells to recruit new blood vessels into the BM [24]. The interaction of MM cells and BM stromal cells also leads to increased secretion of metalloproteases, promoting bone resorption and tumor invasion [25,26].

As the MM cells localize to the BM, they are directly exposed to immune cells [3,27]. Nevertheless, the immune system becomes increasingly impaired as the disease progresses. In fact, loss of the anti-tumor-specific function of T cells is a hallmark of progression from MGUS to MM [28]. This underscores that the evolution of MM is associated with an immunosuppressive microenvironment that fosters immune escape and tumor growth [25,29]. Several mechanisms may contribute to immune escape, including inadequate antigen presentation, resistance to lysis by natural killer cells (NK), and defective immune cells (T, B, NK, and Dendritic cells) [17,27,29,30,31]. Such impairments could be the result of the increased production of myeloma-derived cytokines in the BM milieu, including IL-10, IL-6, and transforming growth factor (TGF)-β [29,30,32]. Indeed, all of these factors can lead to suboptimal tumor-specific immune responses and thereby promote disease progression [29].

## 2. Current Treatment Options for Multiple Myeloma (MM)

An increased understanding of the interactions between malignant plasma cells and the BM microenvironment has led to the identification of new treatment paradigms [17]. The development of novel therapeutic agents, including proteasome inhibitors (PIs) and immunomodulatory drugs (IMiDs), has taken place over the past decade with the aim of improving poor patient outcomes [33]. PIs, such as bortezomib, ixazomib, marizomib, and oprozomib, are designed to disrupt normal degradation of intracellular proteins by the proteasome, thereby leading to cell-cycle arrest, stimulation of apoptosis, and inhibition of angiogenesis [34,35]. IMiDs, such as thalidomide and lenalidomide, stimulate apoptosis of established neovasculature and inhibit angiogenesis and cell-cell adhesion, thereby counteracting the protective effect of the BM milieu [36]. They can also stimulate anti-MM activity by enhancing the immune response against myeloma cells by NK cells [37]. It has also been shown that IMiDs can co-stimulate CD4^+^ and CD8^+^ T cells through phosphorylation of CD28, which, in turn, augments immune responses against MM cells [38].

Another method through which the immune system has been used to exert an anti-MM activity is with hematopoietic stem cell transplantation (SCT). Autologous SCT with high-dose chemotherapy is considered the standard-of-care for newly diagnosed MM patients who are otherwise eligible for transplant [39,40]. Use of the PI and IMiD-based combinations in addition to high-dose melphalan and autologous SCT have been shown to improve the rate of complete response and event-free survival reliably [41]. Allogeneic SCT has been shown to result in durable responses in MM patients who received grafts from HLA-matched sibling donors [42]. Allogeneic SCT offers the advantage of introducing an immune system that has not been negatively influenced by the presence of tumor cells. However, administration of allogeneic immune cells through hematopoietic SCT or donor lymphocyte infusion (DLI) is not uniformly recommended over autologous transplantation for MM due to lack of a survival improvement and a high risk of treatment-related mortality and the risk of developing graft-versus-host disease (GvHD) [40].

The arrival of novel therapeutic agents with the use of SCT has significantly improved treatment outcomes and patient survival. Median survival has increased from two years (in the 1980s and 1990s) to more than five years in those treated in subsequent decades [2,43,44]. Nevertheless, MM remains an incurable disorder due the acquisition of drug-resistant malignant residual disease that can lead to relapse [17], highlighting the need for alternative and more promising therapeutic strategies.

Immunotherapy is an attractive emerging approach for the management of MM. Since immunotherapy is based on generating or augmenting the immune response against cancer cells [45], IMiDs and SCT could both be considered as immunotherapeutic approaches. That said, in this review we will focus on various new immunotherapeutic strategies based on adoptive cell therapy as well as monoclonal antibodies (mAbs). We will also summarize the state of knowledge regarding what targets are currently being employed and what targets are under evaluation for the treatment of MM.

## 3. Monoclonal Antibody (mAb) Therapy

Therapies based on mAbs represent a major advancement in the treatment of MM. Clinical studies have shown promising results for mAb therapy in patients with advanced disease, particularly in combination with other therapeutic agents [46]. Therapeutic mAbs make use of several different mechanisms in order to exert clinical efficacy, each of which is dependent on the ability to activate the immune system. Via one mechanism, mAbs act as a link between tumor cells and immune effector cells, binding to tumor antigens through their hypervariable region and immune cells through their Fc region. This triggers tumor cell death through antibody-dependent cellular cytotoxicity (ADCC). Alternatively, mAbs can activate a cascade of proteolytic enzymes, ultimately resulting in the formation of a terminal lytic complex that ruptures the target cell membrane. This is a mechanism known as complement-dependent cytotoxicity [47]. A more direct effect of mAbs can be through agonist activity: binding and subsequent activation of a cell surface receptor on tumor cells that triggers apoptosis. Conversely, mAbs can also exhibit antagonist activity, in which binding to a cell surface receptor blocks the downstream signaling that is required for cell proliferation or survival, for instance. Finally, conjugation of mAbs to cytotoxic drugs, small interfering RNA, or radioisotopes allows them to serve as carriers, delivering their cargo specifically to the targeted tumor cells rather than systemically [48].

In contrast to mAb therapies that directly target tumor cells, mAbs can alternatively demonstrate anti-tumor effects through targeting of host immune cells. This allows for a specific antibody to be applied to multiple types of cancer. For instance, blockade of a receptor involved in immune checkpoint signaling, PD-1 (see below), has resulted in tumor regression in many different types of cancers [45]. This strategy is known as immune checkpoint blockade and functions by turning off self-tolerance mechanisms: systems in place to prevent auto-immunity that are co-opted by tumor cells to allow for immune escape.

A key challenge in the development of useful mAb therapies is identification of appropriate tumor-specific targets. Targets for mAbs in MM are quite diverse [46] and clinical applications for some mAbs in the management of MM will be discussed below.

## 4. Adoptive Cell Therapy

Adoptive cell therapy (ACT) involves enrichment, ex vivo expansion, and/or modification of autologous or allogeneic lymphocytes followed by infusion into the patient. Successful ACT is determined by the ability of the infused lymphocytes to traffic to the tumor site and to mediate tumor destruction. In the case of T cell therapy, this infusion is often accompanied by administration of the T cell growth factor, IL-2, to enhance expansion of product in vivo. Host lymphodepletion, either by chemotherapy alone or in combination with total-body irradiation, is often recommended to facilitate homeostatic lymphocytic expansion and persistence of the transferred T cells [49,50].

Promising results were reported in 2015 from a phase I clinical trial involving ex vivo stimulation of autologous marrow-infiltrating T cells via immunomagnetic beads (anti-CD3/CD28 beads) and IL-2 followed by infusion into patients after myeloablative therapy. Twenty-five patients with newly diagnosed or relapsed MM were enrolled in this trial. Partial responses or better were observed in 54% of the patients. In addition, the study showed patients who achieved very good partial responses also realized a 90% reduction in tumor burdens. Those patients also showed significantly increased progression-free survival (25.1 months versus 11.8 months; *p* = 0.01), compared to those who did not respond as well. Persistence of myeloma-specific immunity in the BM at one year post-ACT was observed in all enrolled patients, but to a higher level in patients who achieved complete remission [51]

Similarly, adoptive NK cell therapy has been tested in subsets of MM patients. Infusion of haplo-identical NK cells followed by auto-SCT induced near complete remissions in 50% of patients with advanced MM [52]. However, the availability of suitable donors and limited quantities of NK cells for infusions are major challenges to this approach. With this in mind, a recent study tested the feasibility and safety of NK expansion ex vivo before their administration to heavily pre-treated patients with high-risk relapsing MM. Cells were obtained from the myeloma patients themselves or from haplo-identical family donors. Infusion of the expanded NK cells was found to be feasible and safe; however disease progression was not affected in at least 62% of the patients [53].

Allogeneic NK cells expanded from cord blood have also been given in combination with auto-SCT, melphalan, and lenalidomide in a phase I trial of 12 MM patients [54]. That study aimed to use donor-recipient KIR ligand mismatch and NK-cell reactivity to facilitate long-term remission. Results indicate that this approach was safe and feasible, with no serious adverse events reported and no GvHD [54]. Eight patients achieved a near-complete response, and three others had a partial response or very good partial response [54]. It is worth noting that besides the fact there were no control groups in either of the aforementioned studies, NK therapy there was combined with auto-SCT (with or without chemotherapy). That said, these trials suggest that strategies for adoptive NK cell therapy may be successful in the management of MM under the right circumstances, and further development of these therapies may allow them to be as successful as adoptive T cell therapy.

### 4.1. Genetically-Redirected Immune Cells

Caveats that come with adoptive transfer of naturally-occurring, tumor-infiltrating T cells are the requirements for pre-existing tumor-reactive cells that can be isolated, enriched, and expanded ex vivo [49]. The process by which these cells are isolated and expanded can be technically difficult and costly. Genetic engineering of lymphocytes is a huge leap forward for cancer immunotherapy [50], as it enables relatively rapid generation of effector cells with redirected specificity for tumor-associated antigens (TAAs). Thus, T cells can be genetically engineered to express receptors, such as a T cell receptor (TCR) with high recognition specificity for a TAA [55,56]. In this embodiment of ACT, T cells are obtained from the patient and transgenes encoding the desired TCR are introduced into the T cells using viral vectors or other methods. The modified T cells can then recognize the target antigen as a peptide-major histocompatibility complex (MHC) unit. A limitation to this application is that tumor cells frequently downregulate MHC expression in order to escape T cell recognition [50,56]. As well, heterologous pairing with endogenously-expressed TCRs can lead to unanticipated off-target effects [57].

**Figure 2:**
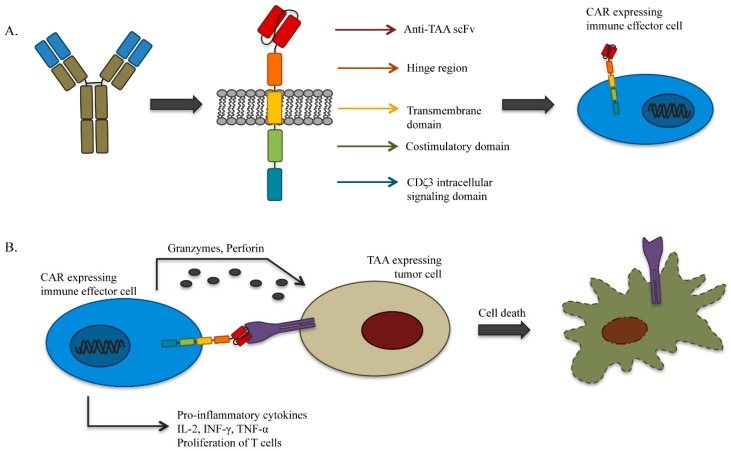
(**A**) A monoclonal antibody can be converted to a chimeric antigen receptor (CAR) through sequencing of the variable light and heavy chains and assembly of those regions into a single-chain variable fragment (scFv). This tumor antigen-specific scFv, combined with signaling regions from the T cell receptor, is then expressed on the surface of an immune effector cell, allowing it to bind to surface antigens on tumor cells; (**B**) binding between the CAR and its TAA triggers the release of cytotoxic granules as well as cytokines, resulting in lysis of the tumor cell and activation of the host immune system.

Chimeric antigen receptor-T cell (CAR-T cell) therapy offers an alternative, MHC-independent, approach to adoptive T cell therapy for MM. Similar to the endpoint in TCR therapy, genetic manipulation of T cells to create CAR-T cells introduces antigen-specific moieties. The selected effector lymphocytes can also be equipped with enhancing properties required for effective tumor elimination [49]. As depicted in Figure 2, CAR-T cells are typically constructed by fusing a single-chain variable fragment (scFv) (derived from a mAb specific for a cell surface TAA) with an intracellular signaling domain. The variable regions from the antibody (the extracellular domain) allow for recognition of MHC-independent structures on the surface of the target tumor cells, while the intracellular co-stimulatory and signaling domains initiate the self-renewal and lytic function of T cells upon antigen binding. Identifying appropriate antigens present on the surface of tumor cells or on their permissive microenvironment but not present on healthy tissues, is a major discriminatory factor for generation of successful CAR-T cell therapy [45,49,58]. Clinical application of broad CAR-T cell-based therapy is hampered by development of ‘on-target, off-tumor’ toxicity resulting from the recognition of the antigen in normal tissues. One method of addressing issues with the safety of CAR-T cell therapies is through insertion of cell-fate control [59,60,61] or ‘suicide’ elements, which allow for the depletion of the infused CAR-T cells in case of adverse events [62].

It is worth noting that CAR therapy is not restricted to T cells; CAR-NK effector cell therapy is another approach under development. An advantage for the use of NK cells over T cells is that NK cells may be less likely to cause cytokine release syndrome, due to a different profile of cytokine production compared to T cells. Additionally, they are less likely to induce GvHD in an allogeneic setting [63]. Thus, NK cells are a potentially safer effector cell population. NK cell lines are also available for use as CAR effector cells, offering the potential for “off-the-shelf” therapies. However, CAR-T cell therapy has entered successfully into clinical trials and shows some promising results, while CAR-NK cells are still limited to pre-clinical studies at this time. Current CAR-based clinical trials for MM will be discussed below in the section of specific targeted antigens.

### 4.2. Dendritic Cell (DC)-Based Vaccines

An alternative to ex vivo expansion of anti-MM effector T cells is the administration of DC vaccines. These vaccines are DCs prepared ex vivo to present tumor-specific peptides that are then delivered back into the patient. These antigen-presenting cells (APCs) have the potential to stimulate a potent anti-tumor T cell response in vivo [64,65].

At least two separate DC-based vaccination approaches have been developed and used for the treatment of MM. In one approach, autologous DCs are pulsed with tumor-derived clonal immunoglobulins (i.e., idiotype). The idiotype (Id)-pulsed autologous DC vaccines are then administered to the patients allowing for recognition by host antibodies and effector T cells, stimulating both the humoral (antibody release) and cellular anti-tumor immune response, respectively [65,66]. This approach is based on the finding that Id-reactive T cells have been detected in the peripheral blood of patients with MGUS and MM [67,68]. However, although such T cells are able to generate anti-Id responses in vitro, antigen presentation appears to be insufficient to invoke effective anti-tumor response in vivo [67,68].

A clinical trial therefore investigated the benefit of using Id-pulsed DCs generated from CD34^+^ hematopoietic stem/progenitor cells in order to enhance Id presentation to host Id-reactive cells. The pulsed DCs showed good tolerability in 11 patients studied with advanced MM, but humoral responses were seen in just three patients and cellular immune response was shown in only four of the 11 patients [66]. A second study likewise investigated stimulation of autologous DCs ex vivo with idiotypic proteins, this time giving the vaccine after autologous SCT. With administration of the vaccine, known clinically as Mylovenge™, a significant improvement in overall survival was reported, however, this was in comparison to historical outcomes as no control arm was included in the trial [69]. Such results therefore need to be confirmed in a more detailed study.

In an alternate approach to DC vaccine therapy in MM, autologous cancer cells are fused to either autologous or allogeneic DCs forming hybridomas, which allow antigen presentation in an MHC-dependent manner with the ability to elicit cytotoxic T cell responses [65]. Rosenblatt and colleagues evaluated this approach in both phase I and phase II clinical trials. In both trials, re-infusion of the patient-derived hybridoma was found not only to be safe, but to induce an immune response as demonstrated by expansion of tumor-reactive T cells and increased production of a tumor-specific antibody [70,71]. The majority of phase I patients had disease stabilization. Combination of this DC therapy with autologous SCT in the phase II trial resulted in cytoreduction of minimal residual disease (MRD), with the majority of patients (78%) achieving very good partial remission or complete remission [71]. Currently, an ongoing clinical trial (NCT01067287) is combining a programmed death-1 (PD-1) blocking antibody with DC/myeloma fusion vaccination with the aim of further improving immune response and patient survival. A randomized phase II trial (BMT CTN 1401) using this approach is currently ongoing in the US targeting patients immediately following an autologous transplant.

## 5. Targets for Immunotherapy in MM

A wide range of antigens may serve as immunotherapeutic targets in MM. Targets currently being studied range from cell surface proteins on MM cells or stromal cells in the BM to non-cellular components in the BM niche, such as growth factors and cytokines [72]. In all cases, viable targets are involved in facilitating tumor progression through a variety of mechanisms, including cell survival, proliferation, angiogenesis, and interactions between MM and BM cells. Ideal targets are those that are: (1) restricted to MM in their expression; and (2) expressed on the majority of MM cells or BM microenvironment [72,73]. Unfortunately, a truly ideal target is difficult to find; each of the objectives discussed below have advantages and disadvantages associated with their use.

### 5.1. Targets on Myeloma Cells

#### 5.1.1. SLAMF7, (CS1, CD319)

SLAMF7 (Signaling lymphocyte activation molecule family member 7), previously known as CS1 (CD2 subset 1), is a cell surface glycoprotein with high expression on malignant plasma cells in most MM patients. It is also expressed on normal plasma cells, NK cells, CD8^+^ T cells, and NK-like T cells (NKT cells) [74]. The exact role of CS1 is not well understood; accumulating evidence suggests that it promotes the adhesion of MM cells to BM stromal cells and mediates tumor cell proliferation and survival [75].

A humanized mAb (elotuzumab) targeting CS1 showed anti-tumor activity in experimental models that was mediated through ADCC [74,76]. In phase I clinical trials for elotuzumab, acceptable toxicities and minimal single agent clinical activity were demonstrated when the drug was administered to patients with refractory or relapsed MM. Stable disease was achieved in 26.5% of patients [77]. Subsequent trials have studied the effect of elotuzumab in combination with other agents especially IMIDs. In a recent phase III clinical trial of 646 patients with refractory or relapsed MM, treatment with elotuzumab in combination with lenalidomide and dexamethasone was compared to lenalidomide and dexamethasone alone. That study showed a significantly higher overall response rate in the elotuzumab group, with a longer progression-free survival [78]. Based on these results, SLAMF7 appears to be a promising candidate for MM immunotherapy.

#### 5.1.2. CD38

CD38 is a cell surface transmembrane glycoprotein that is ubiquitously expressed, though at relatively low levels, on cells of hematopoietic and non-hematopoietic origin. T cells in medullary thymocytes, precursor B cells in the BM, plasma cells, monocytes, and subpopulations of NK cells are some of the hematopoietic cells that express this antigen. It is also present in other tissues including prostate, pancreas, and smooth muscle [79]. CD38 plays a role in cell adhesion and also demonstrates enzymatic activity involved in nucleic acid metabolism [80]. The high expression of CD38 on MM cells makes this molecule an attractive target for therapy [81].

Daratumumab is a humanized monoclonal anti-CD38 antibody that was shown, in pre-clinical studies, to have anti-myeloma activity through ADCC, complement-dependent cytotoxicity, and antibody-dependent phagocytosis [82]. Recently published phase I and phase II studies of daratumumab showed encouraging clinical activity and good tolerance in heavily pretreated patients with refractory myeloma. Daratumumab as a single agent yielded a 36% overall response rate and, in the responder group, 65% remained progression-free over a period of 12 months [83].

A second anti-CD38 mAb, SAR650984 (known clinically as isatuximab), also showed potent anti-myeloma activity in vitro and in vivo [84]. Early clinical trials indicate that SAR650984 is well tolerated, and a response rate of 30% has been reported for single agent use in patients with refractory or relapsed MM [85]. More recent studies have shown even more impressive clinical efficacy when daratumumab or isatuximab was used in combination with lenalidomide or pomildomide or bortezomib [86].

#### 5.1.3. CD40

CD40 is a cell surface transmembrane glycoprotein in the tumor necrosis factor (TNF) superfamily. This antigen is expressed on normal B cells, DCs, and plasma cells. It is also highly expressed on the surface of tumor cells in the majority of MM patients [87,88]. Binding between CD40 and its natural ligand (CD40L) induces cell proliferation and migration via PI3K and NF-κB signaling pathways. In MM, CD40 is thought to promote tumor growth through autocrine IL-6 stimulation and induction of VEGF [89,90,91]. Therefore, inhibition of CD40-CD40L interaction seems a reasonable approach to attempt to exert an anti-myeloma activity.

A mAb against CD40 (SGN-40, known clinically as dacetuzumab) showed cytotoxic activity in MM cell lines and primary cultures through promotion of TNF-induced apoptosis [92]. An early clinical trial of dacetuzumab in patients with advanced MM demonstrated an acceptable safety profile and modest clinical response, with stabilization of disease in 20% of patients [93]. The observation that dacetuzumab-induced cytotoxicity in vitro is enhanced by pre-treatment with lenalidomide [94] resulted in a study evaluating its use in combination with lenalidomide and dexamethasone. A phase I clinical trial of the combination therapy yielded an overall response of 39% in patients with refractory or relapsed MM [95].

A second anti-CD40 mAb, lucatumumab, is a fully humanized antibody demonstrated to have anti-myeloma activity both in vitro and in vivo [96]. Unfortunately, when evaluated in patients with refractory or relapsed MM, lucatumumab showed minimal clinical efficacy [97] and gastrointestinal cytotoxicity was observed [98].

XmAbCD40 is a third humanized anti-CD40 antibody, which demonstrated increased affinity and enhanced cytotoxicity against MM cell lines [99]. Clinical studies of XmAbCD40 have yet to be carried out.

#### 5.1.4. CD138 (Syndecan-1)

CD138 is a membrane protein and a member of the syndecan family of heparan sulfate proteoglycans. It functions as an adhesion molecule, binding to the extracellular matrix (ECM) molecules collagen and fibronectin [100]. It is also involved in cell proliferation [101]. In hematopoietic tissues, CD138 expression is restricted to malignant and differentiated plasma cells [101,102]. CD138 is also expressed in mature epithelial cells [103]. Due to its expression in 100% of MM patients, CD138 is used as a primary diagnostic marker [104].

Membrane-bound CD138 plays a role on tumor progression through mechanisms such as increasing VEGF receptor-2 (VEGFR-2)-mediated angiogenesis [105]. Furthermore, serum levels of shed CD138, a soluble form of the protein, have been shown to correlate with a poor prognosis [106]. Shed CD138 promotes survival and invasion of malignant plasma cells in vivo and is thought to contribute directly to the growth and dissemination of MM cells [107]. Hence, CD138 has been explored as a candidate antigen for antibody-based therapies [108].

Maytansinoid (a microtubule toxin) was used as an immunoconjugate to target CD138 (BT062, known clinically as indatuximab). This compound has shown cytotoxic activity against MM cells using in vitro and in vivo models [109]. In a phase I/II clinical study, monotherapy with BT062 showed modest efficacy [110]. That said, a combination of BT062 with lenalidomide and dexamethasone in patients with refractory or relapsed MM in a separate phase I/II trial resulted in an overall response rate as high as 78% [111].

A pilot clinical trial was undertaken using a second-generation recombinant lentiviral vector to generate anti-CD138 CAR-T cells [112]. Five patients diagnosed with refractory MM were treated. After a follow-up for seven months, four patients were found to have stable disease, and one patient with advanced plasma cell leukemia had a reduction of myeloma cells (from 10.5% to <3%) in the peripheral blood [112]. Data from this study showed that the CAR-T cells homed to the BM. These results suggest that CD138 CAR-T cell therapy for MM is well-tolerated and has potential anti-tumor activity. This research is still underway (on-going phase I/II study (NCT01886976)) [112].

Although targeting CD138 appears to be an attractive approach for MM therapy, it may provide a mechanism for tumor escape due to the existence of CD138-negative MM cells. These cells are found at low frequency, however they have been shown to be drug resistant and, importantly, to possess tumor-propagating activity [113]. As such, anti-CD138 therapies may eventually need to be used in combination with other types of therapy in order to target the full spectrum of MM cells.

#### 5.1.5. CD56 (NCAM1, Leu-19)

CD56 or neuronal cell adhesion molecule (NCAM) is a cell surface glycoprotein, which is a member of the immunoglobulin superfamily. In healthy tissues it is expressed on the surface of neural cells, epithelial cells, NK cells, and a subpopulation of activated T cells [114]. CD56 is also highly expressed on plasma cells from the vast majority of patients with MM [115,116,117]. CD56 is known to mediate cell-cell and cell-matrix interactions [116,117].

HuN901 (IMGN901, known clinically as lorvotuzumab) is a humanized maytansinoid-conjugated mAb that binds with high affinity to CD56. HuN901 has demonstrated significant anti-myeloma activity both in vitro and in a murine xenograft model [118]. Based on these promising preclinical results, lorvotuzumab was evaluated as a single agent in a phase I trial of MM patients. Clinical benefit was observed in 41% of patients, with objective responses lasting between 3 and 20 months [119]. A subsequent study evaluated lorvotuzumab in combination with lenalidomide and dexamethasone (phase I, NCT00991562). Preliminary findings show an overall response rate of 56.4% [119]. However, peripheral neuropathy was identified as the most common dose-limiting toxicity [119], due to CD56 expression in the central and peripheral nervous system [117].

#### 5.1.6. CD74

CD74 is a type II transmembrane protein. It is found primarily on normal B cells, monocytes, macrophages, DCs, and activated T cells [120]. In addition, it is highly expressed in the majority of B-cell malignancies, including MM. CD74 has a role in antigen presentation and MHC class II function [121], as well as in cell proliferation and survival, through the NF-κB signaling pathway [122,123].

The mAb milatuzumab (hLL1) showed promising anti-myeloma activity in vitro [122], leading to its evaluation in a phase I trial in patients with refractory or relapsed MM. This study showed a good safety profile for this therapy, but no objective response was observed at this early phase [124].

CD74 is characterized by rapid internalization, therefore, it is an attractive target for therapies involving the delivery of cytotoxic agents conjugated to mAbs. Pre-clinical studies have evaluated milatuzumab in conjugation with bortezomib, doxorubicin, or dexamethasone. All drug-milatuzumab conjugates were shown be effective in vitro and in vivo, with milatuzumab mediating the intracellular delivery, and thereby enhancing the cytotoxicity of, the other agents [125,126]. A phase I/II study examining the safety and efficacy of milatuzumab-doxorubicin in MM was recently completed (NCT01101594), however the results are yet to be announced.

#### 5.1.7. CD200 (MOX1, MRC, OX-2)

CD200 is type I transmembrane glycoprotein from the immunoglobulin superfamily. It is expressed on normal activated T cells, B cells, and DCs [127]. While CD200 is absent on normal plasma cells, 78% of malignant plasma cells express this antigen. Expression of CD200 on plasma cells is associated with poor prognosis in patients with MM [128]. CD200 is known to play a role in macrophage inhibition [129,130] and it promotes the suppression of immune responses mediated by T cells expressing the corresponding receptor [131].

ALXN6000 is a humanized anti-CD200 mAb that was evaluated in a phase I/II study (NCT00648739) in patients with refractory or relapsed MM. This trial has recently been completed, with results yet to be reported.

#### 5.1.8. CD19

CD19 belongs to the immunoglobulin superfamily and acts as a dominant signaling component of a multi-molecular complex on the surface of mature B cells. CD19 is expressed throughout B cell development, beginning at lineage commitment until differentiation into plasma cells [132]. As a result, it is present on many B cell cancers such as acute lymphocytic leukemia (ALL) and chronic lymphocytic leukemia (CLL), and has been exploited as a target for those malignancies [133].

CD19 is not typically associated with MM [134], however recent reports suggest that this marker may be expressed more frequently in this indication than was previously thought [135]. Furthermore, CD19 has been reported to be expressed on the subset of MM cells that are resistant to current treatments, making it an attractive target for alternative treatment schemas [136].

A CAR T cell strategy targeting CD19 is currently being tested in a phase I clinical trial against MM in combination with autologous transplant for depletion of CD19-negative tumor cells (NCT02135406). Three of the four patients treated had evaluable outcomes; all responded in some degree to the combination therapy. One patient demonstrated a complete response and maintained a MRD negative status for almost one year [137]. This preliminary data from this ongoing trial suggests that CD19 CAR T cells are safe and may have efficacy for the treatment of MM.

#### 5.1.9. BCMA (TNFRSF17, CD269)

B cell maturation antigen (BCMA) is a member of the TNF superfamily and binds to various TNF cytokines such as the B-cell activating factor (BAFF) and a proliferation-inducing ligand (APRIL) [138,139,140]. This type I transmembrane protein is normally expressed on plasma cells and other mature B cells, where it plays a role in long-term survival [141]. Of note for MM therapy, BCMA is also expressed on the majority of malignant plasma cells, and its lineage-restricted expression makes it a good target for immunotherapy [142,143,144,145,146].

Preclinical studies of a humanized anti-BCMA antibody-drug conjugate J6M0-mcMMAF (known clinically as GSK2857916) identified two mechanisms of cytotoxicity (recruitment of macrophages and ADCC) in response to administration in mouse models of MM [147]. Rapid tumor elimination but a sparing of BM stromal cells, peripheral blood mononuclear cells (PBMCs), and NK cells was seen, supporting initiation of a phase I clinical trial. This trial is currently recruiting patients with relapsed/refractory MM (NCT02064387).

A second method under investigation for targeting BCMA is CAR T cells. This strategy has been undertaken by several groups, and is currently being evaluated in four open clinical trials (NCT02546167, NCT02215967, NCT02658929, NCT02786511). In one study, BCMA CAR T cells, in combination with cyclophosphamide and fludarabine, were administered at varying doses to 12 patients with advanced MM [148]. At low doses, toxicities were found to be mild and responses modest [148]. Toxicities were more pronounced at higher doses (9 × 10^6^ CAR T cells/kg) and included cytokine release syndrome, but efficacy was similarly enhanced, with bone marrow plasma cells in both patients treated at this dose decreasing from 80%–90% to 0% [148]. Other phase I trials for BCMA CARs are currently recruiting patients.

BI 836909 is a bi-specific T-cell engager (BiTE) that binds CD3ε on T-cells and BCMA on plasma cells. In preclinical models, BI 836909 has demonstrated effective anti-myeloma activity [149]. A phase I clinical trial is currently running using this compound; results are yet to be announced (NCT02514239).

#### 5.1.10. Cancer Testis Antigens (GAGE Family, LAGE, MAGE Family, NY-ESO-1, SSX Genes, etc.)

Cancer testis antigens (CTAs) are known to be upregulated on a number of cancers, including MM. Their expression in normal tissues is restricted to the testis, an immune-privileged site, and their expression on malignant cells is shared between multiple tumor types, where they are thought to play a role in tumorigenesis. Several CTAs, such as MAGE-C2 and NY-ESO-1, have been demonstrated to be highly immunogenic: spontaneous patient immune responses have been documented against these targets [150,151]. Additionally, CTA expression has been found to be strongly linked to patient outcomes in MM, where the presence of CTAs is associated with a more malignant phenotype [152,153]. Taken together, this provides strong rationale for CTAs as targets for MM immunotherapy.

Expression of a number of CTAs has been characterized in MM cells [154,155]. NY-ESO-1 has been shown to be expressed on cells from about 13% of MM patients, and SSX family members such as SSX1 and SSX4 are known to be expressed in more than 20% of MM patient samples, but not in MGUS samples [156]. This non-universal expression across MM patients highlights the need to develop therapies against multiple CTAs. For instance, MAGE-C1/CT7, MAGE-A3/6, and LAGE-1 have been shown to, as a group, be expressed on cells in about 85% of MM cases, thus these are attractive targets for therapy [155].

A MAGE-A3 vaccine was studied in a phase II clinical trial in which MM patient T cells were stimulated in vivo with MAGE-A3 peptide vaccination, and subsequently apheresed and expanded ex vivo prior to re-infusion [157]. Patients also underwent auto-SCT and melphalan therapy, and received five additional MAGE-A3 immunizations following T cell infusion [157]. Vaccine-specific, cytokine-producing T cells were detected in 76% of patients treated, demonstrating the high potential of this strategy to stimulate immune response in MM patients [157]. Anti-NY-ESO-1/LAGE therapy has also been studied in a phase I/II trial of TCR-transduced T cells in 20 MM patients [158]. The infused T cells were shown to expand, persist, and exhibit cytotoxicity, resulting in complete or near-complete responses in 70% of patients [158]. All subsequent disease progression was found to be a result of antigen escape or lack of T cell persistence, and no adverse events greater than grade 3 were reported [158].

Other phase I, II, and III trials are currently underway examining peptide vaccines (NCT00090493, NCT01380145), DC vaccines (NCT01995708), and T cell therapies (NCT01892293, NCT01352286, NCT02457650, NCT02291848) for MAGE-A3, NY-ESO-1, LAGE-1, and other CTAs. These studies are currently recruiting, active, or awaiting study results.

#### 5.1.11. GRP78

The glucose-regulated protein GRP78 resides in the endoplasmic reticulum (ER) in normal cells. It facilitates protein assembly and regulates ER stress signaling, and, therefore, is known as the ER-stress gatekeeper [159]. An isoform of GRP78 is expressed on the surface of a majority of primary MM specimens and cell lines [160,161]. GRP78 promotes tumor cell survival, angiogenesis and metastasis [159,160]. It has been reported that expression of GRP78 is associated with resistance to bortezomib [162,163] and BRAF inhibitors [164].

PAT-SM6 is a human monoclonal IgM antibody that targets the cancer-specific isoform of GRP78. PAT-SM6 has been shown to bind to the GRP78 isoform on the surface of MM cells, but has not been shown to react against normal hematopoietic tissue, including plasma cells [165]. In preclinical models, PAT-SM6 has been shown to induce significant cytotoxicity against MM [165,166]. At low doses, PAT-SM6 has also shown a favorable safety profile in patients with recurrent “in-transit” cutaneous melanoma [166]. In a dose-escalating study, Rasche et al. showed that PAT-SM6 is well-tolerated, but with modest clinical efficacy in 12 patients with relapsed and refractory MM [161]. Together, these studies allow an opportunity for further trials exploring the combination of PAT-SM6 with existing therapies.

### 5.2. Targets in the Microenvironment

#### 5.2.1. IL-6

IL-6 is a cytokine that is involved in several pathological conditions, particularly inflammatory disorders. IL-6 is expressed by BM stem cells and binds to the IL-6 receptor (IL-6R) on myeloma cells thereby stimulating their proliferation and survival [167]. It has been recognized as a key molecule in the development of MM, bringing it to the forefront as a cytokine that may be targetable for therapeutic purposes [168].

Siltuximab (CNTO328) is a chimeric anti-IL-6 mAb that was shown to enhance the cytotoxic effect of melphalan, dexamethasone, or bortezomib with dexamethasone in MM cell lines and in cells from refractory MM patients [169,170]. A phase I study demonstrated the safety and tolerability of siltuximab with modest efficacy (complete response in two of 13 patients) [171]. Siltuximab either alone, or in combination with dexamethasone, was evaluated in a phase II trial involving refractory or relapsed MM patients [172]. That study reported no response to siltuximab alone, but an overall response rate of 17% in patients receiving the combination therapy [172]. However, when a combination of siltuximab and bortezomib was compared to bortezomib alone, the addition of siltuximab failed to improve progression-free survival and overall survival in a phase II trial [173]. Several trials evaluating IL-6 blockade in combination with other agents in MM are currently ongoing (NCT00911859, NCT00401843, and NCT01266811).

#### 5.2.2. PD-1/PD-L1

PD-1 is a member of the B7-CD28 family of receptors. It is expressed on antigen-activated and exhausted T and B cells, where it functions as an immune checkpoint. When PD-1 binds to its cognate ligand (PD-L1) an inhibition of the immune response occurs, resulting in immune tolerance and prevention of normal tissue damage [174,175].

The ligand for PD-1, PD-L1, is expressed on the surface of APCs [174]. While PD-L1 is absent on normal epithelial tissues, aberrant expression has been reported on several solid tumors and is associated with a poor prognosis [176]. MM cells have been shown to express PD-L1, while normal plasma cells do not [177]. In addition, PD-1 was found to be expressed on NK cells derived from MM patients, but not on NK cells originating from healthy individuals [178]. Furthermore, it was shown that BM stromal cells enhance upregulation of PD-L1 on MM cells, resulting in enhanced immune escape and increased aggression of myeloma cells [179]. In light of this, targeting of PD-1 on T cells, or PD-L1 on tumor cells, has gained interest as an immunotherapy-based treatment schema for MM with the goal of turning off acquired tumor immune tolerance.

The primary mechanism of action of PD-1/PD-L1 inhibitors is via improved survival of T cells, however some evidence also suggests their involvement in the activation of NK cells [178,180]. Immune checkpoint inhibitors targeting the PD-1/PD-L1 axis (nivolumab, pembrolizumab, and pidilizumab) have been tested in treatment of various types of tumors. In MM, a phase I study with nivolumab reported that 67% of patients remained with stable disease, though no objective responses were seen [181]. The safety and efficacy of pembrolizumab, in combination with pomalidomide, has been evaluated in a phase II trial involving patients with refractory or relapsed MM [182]. Preliminary results reported 50% objective responses, including near complete and very good partial responses [182]. Pidilizumab (CT-011) was investigated in a phase I study in patients with various hematologic malignancies, including MM after chemotherapy and/or SCT [183]. In that study, clinical efficacy was observed in 33% of the patients [183]. The combination of CT-011 with a DC/myeloma fusion vaccine is also being investigated, with a phase II study currently underway (NCT01067287).

Several clinical studies are also underway to investigate the use of inhibitors of the PD-1/PD-L1 axis in various combinations with IMiDs for the treatment of MM (NCT02036502, NCT02289222, NCT02576977, NCT02077959, NCT02331368, NCT02579863 and NCT02431208, for example).

#### 5.2.3. KIR

Killer immunoglobulin-like receptor (KIR) is a class of inhibitory receptors expressed by NK cells, a minority of CD8^+^ T cells, and rarely CD4^+^ T cells [184,185]. These receptors are key inhibitors of NK cell activity when they bind to human leucocyte antigen-1 (HLA-1) molecules [186]. As MM cells overexpress HLA-1 molecules, KIR are of interest as potential targets for immunotherapy [187].

The fully human mAb (IPH2101) blocks binding of KIRs to their ligands, facilitating activation of NK cells and, potentially, enhanced tumor destruction. IPH2101 was shown to augment cytotoxic activity of NK cells against myeloma cells in vitro and in vivo [188]. Monotherapy of IPH2101 has been investigated in patients with refractory or relapsed MM in a phase I trial [189]. That study showed that the mAb is safe to administer, but no objective responses were achieved [189]. A combination of IPH2101 with lenalidomide was also evaluated in a phase I trial of 15 MM patients [190]. Although an objective response was observed in five patients, five serious adverse events were also reported [190]. Other trials evaluating IPH2101 (alone or in combination with lenalidomide) in patients with smoldering MM or relapsed MM have been completed, and results are pending (NCT01222286 and NCT00999830).

#### 5.2.4. VEGF

VEGF is a key cytokine produced by cells in order to stimulate vasculature and angiogenesis in their microenvironment. It has been implicated in promotion of tumor growth and survival in various malignancies. Increased expression and secretion of VEGF by myeloma cells has been reported [191].

Bevacizumab is a humanized anti-VEGF mAb that binds to soluble VEGF and blocks it from interacting with its receptors. This strategy prevents formation of neovasculature, and has been evaluated extensively in various solid tumors [192]. In MM, bevacizumab has been studied in combination with dexamethasone and lenalidomide and was found to not significantly increase outcomes in comparison to dexamethasone and lenalidomide alone [193]. Similarly, when bevacizumab was combined with thalidomide in a phase II trial, results were comparable between combination therapy and thalidomide monotherapy [194]. Furthermore, the addition of bevacizumab to bortezomib therapy in refectory or relapsed MM patients did not result in significant improvements in patient outcomes, and patients treated with the combination showed more adverse events than patients treated with bortezomib alone [195], warranting caution and close monitoring of recipients.

#### 5.2.5. BAFF/APRIL

BAFF and APRIL are cytokines of the TNF superfamily. They are expressed by normal BM stem cells and osteoclasts. BAFF/APRIL signaling is involved in normal B-cell development [196].

Both BAFF and APRIL have been found to have increased expression levels in MM, and they have been implicated as important players in the interaction between MM cells and the BM microenvironment. The level of BAFF or APRIL in the marrow of MM patients is positively correlated with disease progression [197,198]. Both factors are known to promote viability and proliferation of myeloma cells [199].

The fully human anti-BAFF mAb, tabalumab, has been demonstrated to have neutralizing activity against both membrane-bound and soluble BAFF [200]. In animal models for MM, tabalumab was shown to confer survival advantage with a reduced disease burden [143]. A phase I study evaluating tabalumab combined with bortezomib showed partial responses in 45% of refractory or relapsed MM patients [201]. At least three clinical trials in MM are currently underway to evaluate tabalumab, either alone or in combination with dexamethasone and bortezomib (NCT00689507, NCT01556438, and NCT01602224).

A second agent, atacicept, is a recombinant fusion protein that binds to and inactivates both BAFF and APRIL in their soluble form to inhibit signaling [202]. It has shown promising anti-myeloma activity both in vitro and in vivo [203]. A phase I study of atacicept in refractory or relapsed MM has shown disease stabilization in several patients, but a lack of partial or complete responses [204].

## 6. Summary and Future Directions

MM progression relies on suppression of the immune response. The reversal of immune suppression could potentially enhance intrinsic anti-myeloma activity. This provides a strong rationale for the ongoing exploration of immunotherapies for MM [205]. The ideal immunotherapy should have the ability to overcome the effects of an immunosuppressive microenvironment, demonstrate anti-tumor activity, and improve patient outcomes while maintaining an acceptable toxicity profile. With this in mind, various immunotherapeutic approaches have been developed, including IMiDs, SCT, mAbs, vaccines, and adoptive T cell therapy. These therapies have already started shifting the conceptual and pragmatic paradigms in the management of MM.

Perhaps the most attractive of the strategies discussed here are mAb therapies and CAR-T cell therapy. Although most of mAbs showed significant anti-myeloma activity in pre-clinical studies, dramatic clinical responses are fairly rare with single-agent therapies. Pre-clinical and clinical evidence, however, suggests that mAbs are likely to act synergistically when combined with other therapies; including traditional therapies (e.g., dexamethasone), IMiDs (e.g., thalidomide, lenalidomide), or PI (e.g., bortezomib). Indeed, incorporation of mAbs into combination therapies has been shown to overcome disease that is otherwise refractory to the single agent treatment. These results provide a rationale for the study of multi-agent therapies in future clinical trials.

CAR-T cell therapy provides a “living drug” and, currently, is one of the approaches at the forefront of cancer immunotherapy. However, due to the lack of (known) targets that are expressed only on tumor cells, CAR-T cells may induce adverse cytotoxic activities as a result of their potent action. Several strategies have been proposed to mediate this, including insertion of a suicide element into the vectors, or inclusion of a second inhibitory CAR with specificity for antigens expressed on normal but not tumor cells [58].

Obstacles in the way of developing an ideal immunotherapy for MM are primarily the heterogeneity of the disease and difficulties in identifying an “ideal target” that is expressed exclusively by the malignant cells. Because malignant plasma cells are shielded by a tumor microenvironment that supports their aggressive growth, targeting this environment also seems a reasonable approach. As presented in this review, a number of primary candidates, i.e., targets on either the myeloma cells or the supporting microenvironment, have already been identified. Clinical studies have shown that not all of these are viable candidates, and therefore, efforts are underway to determine which ones are valuable with regard to their clinical efficacy and tolerability. It is important to note that most therapies that have shown clinical efficacy thus far have been evaluated in patients with refractory or relapsed MM, a patient group that is the most difficult to treat. Therefore, these agents may be even more successful in newly diagnosed patients, and may represent valuable tools for the management of all stages of MM in the future.
